# Exploratory factor analysis determines latent factors in Guillain–Barré syndrome

**DOI:** 10.1038/s41598-022-26422-5

**Published:** 2022-12-17

**Authors:** Seiichi Omura, Kazuaki Shimizu, Motoi Kuwahara, Miyuki Morikawa-Urase, Susumu Kusunoki, Ikuo Tsunoda

**Affiliations:** 1 Department of Microbiology, Kindai University Faculty of Medicine, 377-2 Ohnohigashi, Osakasayama, Osaka 589-8511 Japan; 2 Department of Neurology, Kindai University Faculty of Medicine, 377-2 Ohnohigashi, Osakasayama, Osaka 589-8511 Japan; 3 Department of Psychology, Faculty of Sociology, Kansai University, 3-3-35 Yamate-Cho, Suita, Osaka 564-8680 Japan

**Keywords:** Neuroimmunology, Autoimmune diseases, Peripheral neuropathies, Computational biology and bioinformatics

## Abstract

Exploratory factor analysis (EFA) has been developed as a powerful statistical procedure in psychological research. EFA’s purpose is to identify the nature and number of latent constructs (= factors) underlying a set of observed variables. Since the research goal of EFA is to determine what causes the observed responses, EFA is ideal for hypothesis-based studies, such as identifying the number and nature of latent factors (e.g., cause, risk factors, etc.). However, the application of EFA in the biomedical field has been limited. Guillain–Barré syndrome (GBS) is peripheral neuropathy, in which the presence of antibodies to glycolipids has been associated with clinical signs. Although the precise mechanism for the generation of anti-glycolipid antibodies is unclear, we hypothesized that latent factors, such as distinct autoantigens and microbes, could induce different sets of anti-glycolipid antibodies in subsets of GBS patients. Using 55 glycolipid antibody titers from 100 GBS and 30 control sera obtained by glycoarray, we conducted EFA and extracted four factors related to neuroantigens and one potentially suppressive factor, each of which was composed of the distinct set of anti-glycolipid antibodies. The four groups of anti-glycolipid antibodies categorized by unsupervised EFA were consistent with experimental and clinical findings reported previously. Therefore, we proved that unsupervised EFA could be applied to biomedical data to extract latent factors. Applying EFA for other biomedical big data may elucidate latent factors of other diseases with unknown causes or suppressing/exacerbating factors, including COVID-19.

## Introduction

Guillain–Barré syndrome (GBS) is an acute immune-mediated neuropathy in the peripheral nervous system (PNS) with symmetrical areflexia and weakness of the limbs^1,2^. Antibodies to glycolipids, as well as mixtures of two different glycolipids, have been detected in sera from GBS patients. Some individual anti-glycolipid antibodies can be useful diagnostic markers and have been suggested to play pathogenic roles, since these antibodies were often associated with specific clinical signs/symptoms^3^. GBS has often been preceded by infections with microbes such as *Campylobacter jejuni*^4^. Since molecular mimicry between some microbes and neuroantigens of the PNS has been demonstrated experimentally, induction of the anti-microbial antibodies that cross-react with neuronal glycolipids in the GBS has been proposed to explain the antibody-mediated PNS damages.

GBS is a heterogenous disease; different microbial infections and clinical signs/symptoms have been associated with distinct anti-glycolipid antibody seropositivity. However, it is unclear how many target antigens (or the triggering microbes)^5^ exist and whether a set of multiple glycolipid antibodies can be induced by one common factor (e.g., particular microbe or autoantigen). Combinatorial glycoarray is a method to titrate immunoglobulin (Ig) G and IgM antibodies against ten glycolipids [GM1, GM2, GD1a, GD1b, GQ1b, GalNAc-GD1a, LM1, galactocerebroside (G-C), asialo-GM1 (GA1) and sulfatide (Sulfa)] and 45 combinations of the two different glycolipids (total 55 glycolipid antibodies, Figure S1a) in GBS^1,6^. Previously, glycoarray has been used to associate between each glycolipid antibody and specific clinical signs/symptoms^7^ by two-way comparison with control samples. However, no studies have conducted to analyze “a set of” glycolipid antibody data (multiple antibody data altogether). In theory, instead of an analysis of each glycolipid antibody titer, multivariate analysis of a panel of glycolipid antibody titers could provide new insight into the pathogenicity of GBS, for example, regarding a set of glycolipid antibodies as one factor.

For multivariate big data analysis, principal component analysis (PCA) and exploratory factor analysis (EFA) are often used^8^. PCA is intended to reduce data; the principal components extracted in a PCA are not latent constructs (= factor), but PCA examines the total variance of all variables. Since PCA is not based on a hypothesized model, the components extracted from PCA cannot be considered latent factors, thereby limiting the usefulness of PCA. On the other hand, EFA’s purpose is to identify latent constructs underlying a set of manifest variables; EFA is used when the research goal is to identify the nature and number of common factors among the variables.

**Figure 1:**
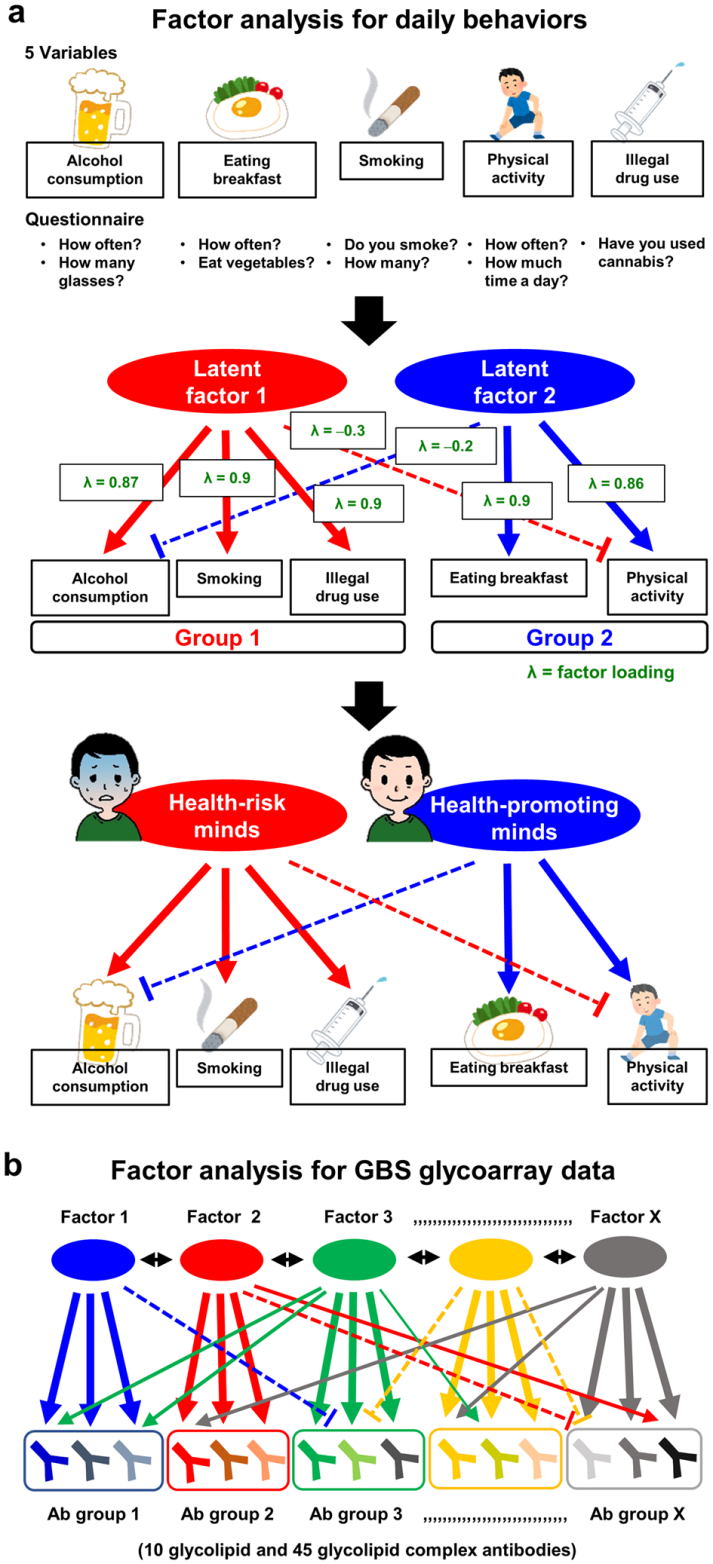
Exploratory factor analysis (EFA)’s purpose is to identify latent factors underlying a set of manifest variables; EFA is used when the research goal is to identify the nature and number of common factors among the variables. (**a**) An example of EFA in the psychology field to identify latent factors underlying daily behaviors. First, prepare a questionnaire listing the items (behaviors): alcohol consumption, eating breakfast, smoking, physical activity, and illegal drug use, asking questions such as how often one drinks alcohol, eats breakfast, or smokes. Based on big multiple data from many people, EFA of the questionnaire data results in grouping these variables (behaviors) and identifies the number of latent factors underlying the behaviors. Here, EFA clusters five behaviors into two groups (Groups 1 and 2) based on factor loading (λ ≥ 0.5, good association; and λ < 0, potential negative association), and extracts Latent factors 1 and 2. From the items (behaviors) in each group, the nature of Latent factors 1 and 2 are likely health-risk and health-promoting mindsets, respectively. (**b**) Hypothetical model of EFA using glycoarray data from patients with Guillain–Barré syndrome (GBS). In the current study, we hypothesized that several factors (Factors 1 to X), such as distinct microbial infection and autoantigen sensitization, may induce unique sets of antibody (Ab) groups (Ab groups 1 to X). If this is the case, EFA of the total 55 antibody data (10 anti-glycolipid and 45 anti-glycolipid complex antibodies) will determine the number (n = X) of factors as well as distinct sets of antibodies that are associated with each factor. By assessing the target antigens of glycolipid antibodies clustered into one group, we would estimate the nature of each factor; i.e., Ab group 1 is composed of three bluish antibodies, associating with the latent blue Factor 1. There can be associations between factors ( ↔) and positive ( →) and negative (—ǀ) associations of one factor to several Ab groups.

EFA has been one of the most widely used statistical procedures in psychological research^9^. For example, to identify latent factors underlying daily behaviors, psychologists use a questionnaire listing the behaviors: alcohol consumption, eating breakfast, smoking, physical activity, and illegal drug use, asking questions such as how often one drinks alcohol, eats breakfast, or smoke (Fig. 1a). Following obtaining multiple data from many people, EFA of the data from the questionnaire results in grouping these variables and identify the number of latent factors underlying the behaviors. In this example (Fig. 1a), EFA classifies five behaviors into two groups (Groups 1 and 2), based on factor loading and extracts Latent factors 1 and 2. From the items (behaviors) in each group, psychologists can assume the nature of the Latent factors 1 and 2 as health-risk and health-promoting mindsets, respectively.

EFA is rarely used in other fields, including biomedical science. In this study, we hypothesized that, upon application of EFA on glycoarray data of GBS patients, several factors, which can be distinct microbial infections, hosts’ genetic factors, etc., could be extracted and associated with the induction of sets of glycolipid antibodies. Figure 1b showed our hypothetical model of EFA using glycoarray data from patients with GBS. If several factors (Factors 1 to X), such as microbial infection and autoantigen sensitization, induce unique sets of antibody (Ab) groups (Ab groups 1 to X), EFA of the total 55 anti-glycolipid antibody data will determine the number (n = X) of factors as well as distinct sets of antibodies that are associated with each factor. By assessing the target antigens of glycolipid antibodies that clustered into one group, we would estimate the nature of each factor, i.e., Ab group 1 is composed of three bluish antibodies, associating with the latent blue Factor 1. Here, there can be associations between factors ( ↔) and positive ( →) and negative (―|) associations of one factor to several Ab groups.

**Figure 2:**
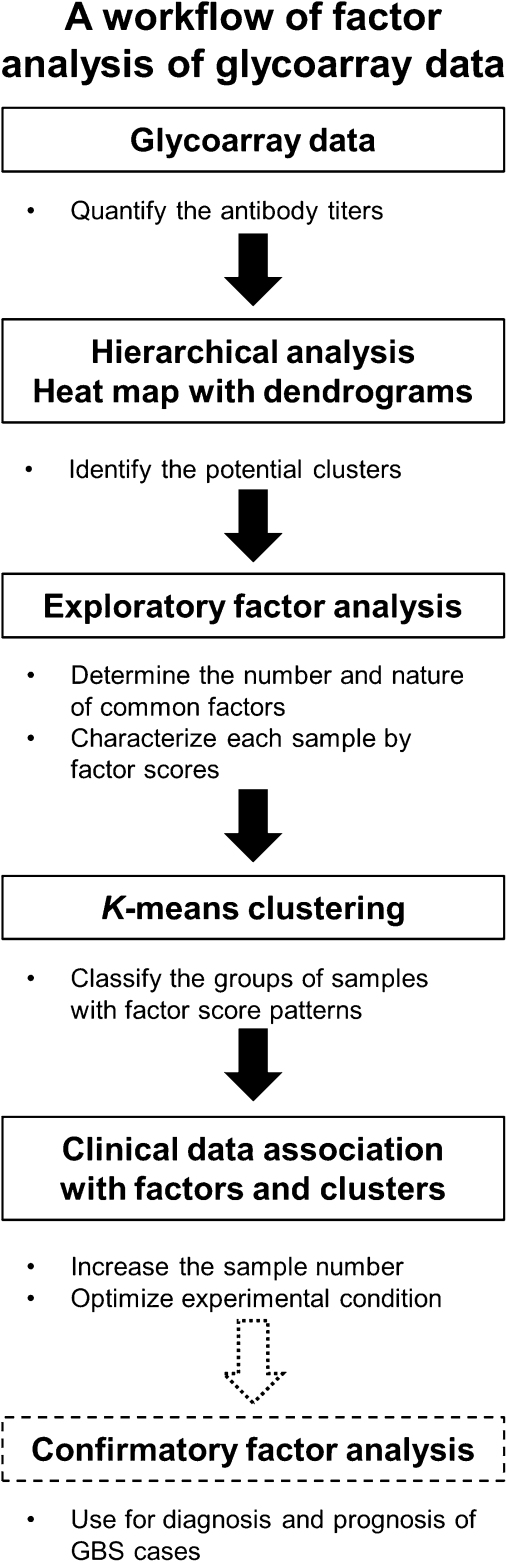
A workflow of factor analysis of glycoarray data from GBS patients. Using glycoarray data, we quantified the total 55 glycolipid antibody titers of 100 GBS and 30 healthy control serum samples. As a hierarchical analysis, we drew a heat map with dendrograms based on similarities of anti-glycolipid antibody titer patterns among samples, identifying the potential clustering of a set of anti-glycolipid antibodies. Using exploratory factor analysis, we identified the number and nature of common latent factors, each of which was associated with a distinct set of anti-glycolipid antibodies, and characterized individual samples by the factor scores of the common factors. Using *k*-means clustering based on the factor scores of individual samples, we classified GBS samples into several clusters with distinct factor score patterns. We associated clinical data with the factors and the patient clusters. In the future, by increasing the number of samples and optimizing the glycoarray condition, confirmatory factor analysis will be applicable for the diagnostic or prognostic purpose of GBS cases.

In this manuscript, we found that PCA did not distinguish GBS and healthy control (HC) samples, using antibody titer data of the glycoarray (Tables S1 and S2)^10^. Thus, we conducted EFA according to a workflow (Fig. 2) and were able to determine four latent factors associated with neuroantigens in GBS, which were consistent with findings reported experimentally and clinically. In addition, EFA extracted one potentially suppressive factor, which has not been reported previously. Here, we proved that unsupervised EFA could be applied to biomedical data to extract latent factors. Thus, applying EFA to other biomedical big data may also elucidate latent factors of other diseases with unknown causes or suppressing/exacerbating factors.

## Results

### PCA of IgG glycoarray data does not distinguish GBS and HC samples

Using combinatorial glycoarray, we titrated IgG and IgM antibodies against 10 individual glycolipids and 45 glycolipid complexes (total 55 glycolipid antibodies) in patients with GBS (n = 100). We diagnosed^11^ and classified GBS patients^12^ into three groups: acute inflammatory demyelinating polyneuropathy (AIDP, n = 37), acute motor axonal neuropathy (AMAN, n = 23), and “unclassified” (n = 33) (Table S3), or into five groups: normal (N), primary demyelinating (D), primary axonal (A), inexcitable (I), and equivocal (E)^13^. We were not able to categorize seven patients (“Other GBS”) since their substantial electrophysiological data were not available. The clinical characteristics of the patients were shown in Table S3^1^.

**Figure 3:**
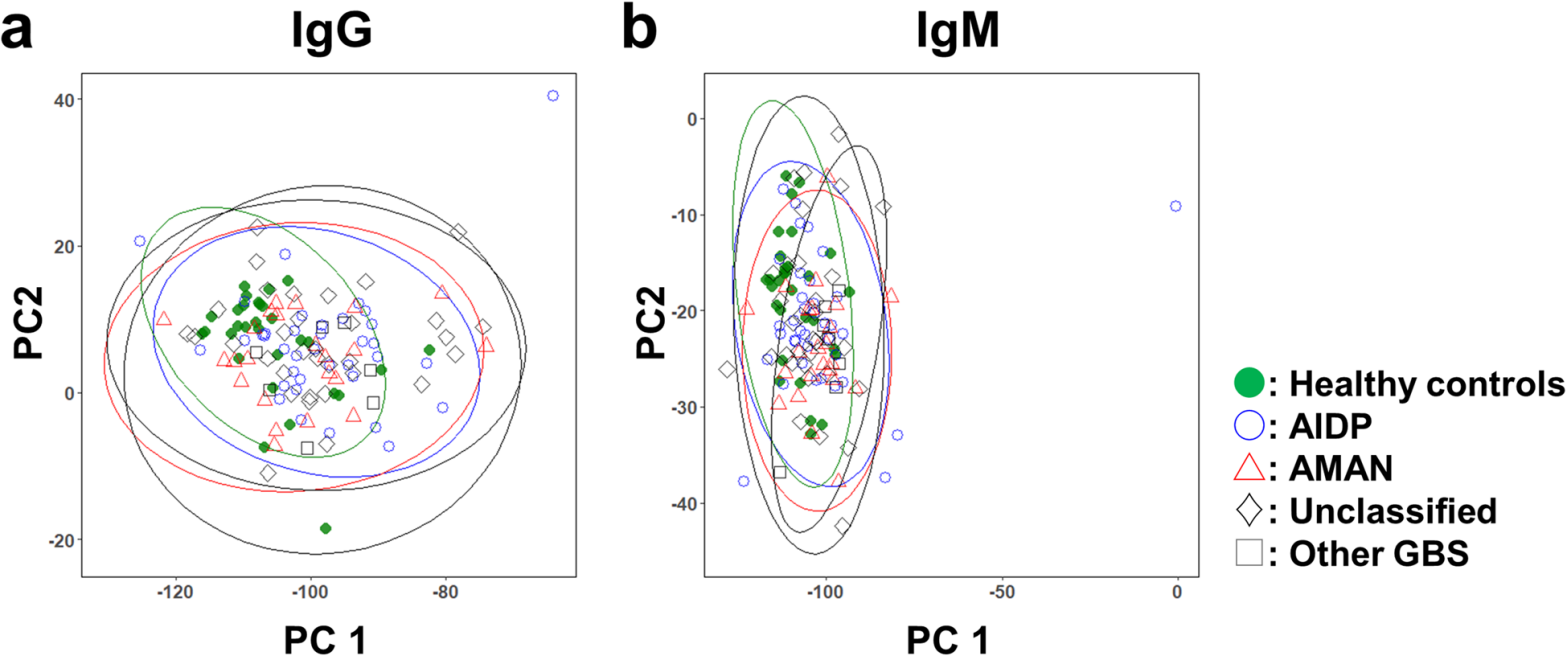
Principal component analysis (PCA) of anti-glycolipid IgG and IgM antibody data from 100 GBS samples, which were composed of 37 acute inflammatory demyelinating polyneuropathy (AIDP), 23 acute motor axonal neuropathy (AMAN), 33 “Unclassified”, and seven “Other GBS” samples, and 30 healthy control (HC) samples. (**a**) PCA of IgG glycoarray data did not separate distinct populations among the GBS subgroup and HC samples by principal component (PC) 1 or PC2 values. An ellipse of the 99% confidence interval drawn for each GBS subgroup or HC group showed the presence of outliers did not explain the failure of the separation as a distinct population by PCA. (**b**) PCA of IgM glycoarray data also did not separate them.

Since we detected various levels of IgG antibodies against glycolipids and glycolipid complexes in GBS samples, we tested whether PCA of the glycolipid IgG antibody titers could separate the samples between the GBS versus HC samples, as well as based on the GBS classification (Fig. 3a). PCA did not separate them between the GBS versus HC samples, or based on the GBS classification by principal component (PC) 1 or PC2 values.

### Hierarchic analysis of IgG glycoarray data suggests clustering of antibodies based on glycolipid antigens, but not GBS subtypes

**Figure 4:**
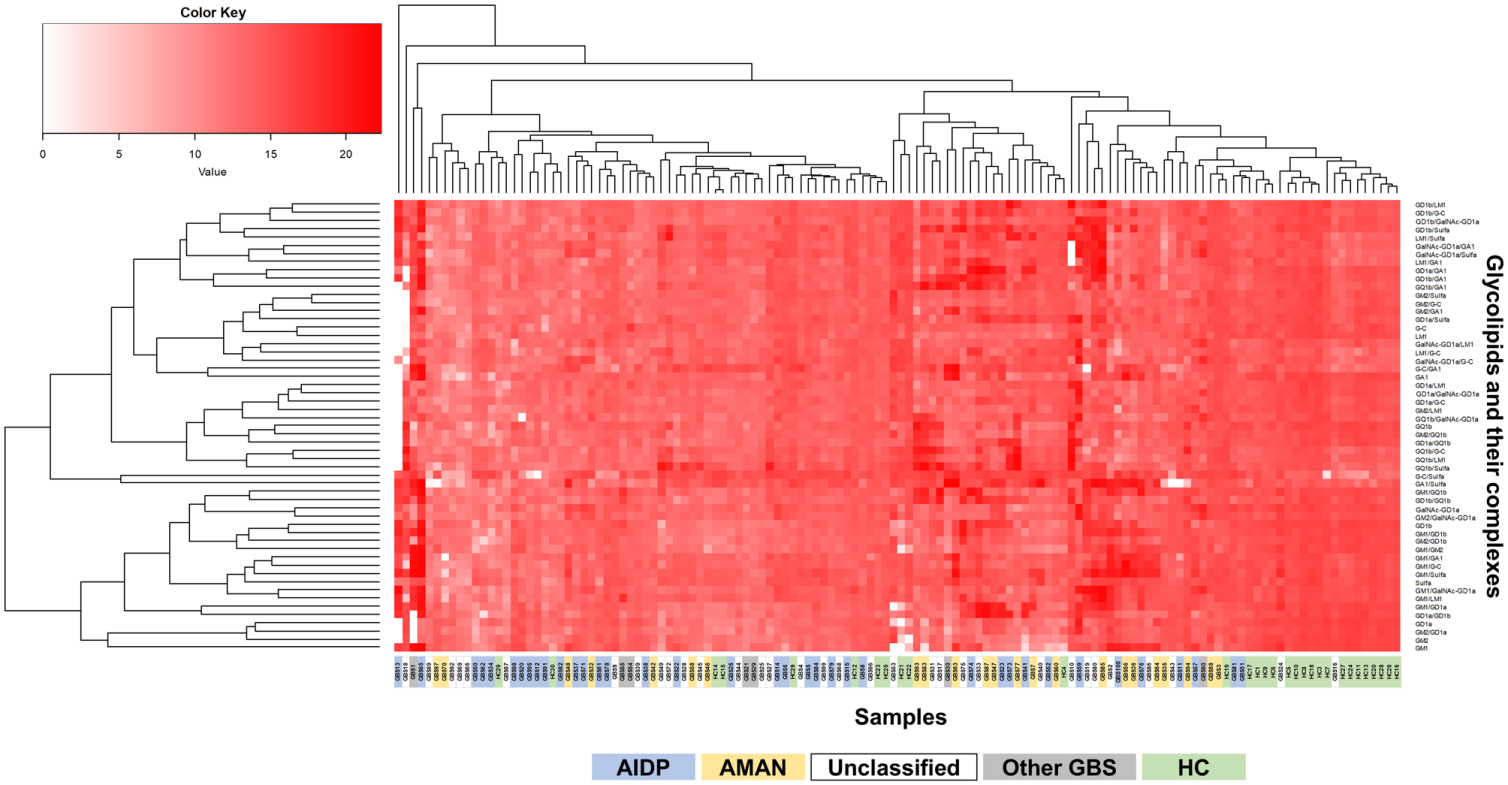
A heat map of serum glycolipid IgG antibody titers in GBS subtypes and HC. We quantified 10 glycolipid and 45 glycolipid complex antibodies by IgG glycoarray and aligned individual samples, using hierarchical clustering. Each row represents glycolipid antibody titers against a single glycolipid antigen, and each column represents a single serum sample. Red density corresponds to the antibody titer level; white indicates the absence of the antibody. A dendrogram at the top is the hierarchical clustering based on antibody titer patterns of individual serum samples. Color bars at the bottom of the heat map indicate that the samples from AIDP, blue; AMAN, orange; “Unclassified,” white; “Other GBS,” gray; and HC, green, showing the dispersal of GBS subgroups and HC samples. This analysis did not segregate the GBS and HC samples into two distinct groups based on the antibody titers, although a dendrogram (at the top of the heat map) based on the patterns of antibody titers showed that the three clusters at the right side of the heat map contained 19 of 30 HC samples (and 8 of 100 GBS samples). We also did not find the segregation of patients’ samples based on GBS subgroups, although several sample groups composed of two to five samples in the same GBS subgroup showed similar patterns of antibody titers. Thus, hierarchic clustering did not distinguish samples between GBS versus HC samples or among GBS subtypes. On the other hand, a dendrogram at the left of the heat map, which is the hierarchical clustering of the 55 glycolipid antibody titers, showing the possible clustering based on a distinct set of antibodies. For example, the top small cluster composed of five antibodies contained four GD1b complex antibodies [i.e., GD1b/LM1, GD1b/GalNAc-GD1a, GD1b/galactocerebroside (G-C), and GD1b/sulfatide (Sulfa)]; the very bottom big cluster composed of 20 antibodies contained GM1 and all nine GM1 complex antibodies. Thus, the groups of glycolipid and/or glycolipid complex antibodies may be composed of clusters.

To investigate whether 1) the GBS subgroups (AIDP, AMAN, “Unclassified,” and “Other GBS”) and HC had distinct patterns of glycolipid antibody titers (i.e., clustering based on the disease subtypes) and 2) a set of glycolipid antibodies showed similar patterns of their levels, we performed hierarchical clustering analysis with a heat map, using 55 anti-glycolipid antibody titers of the GBS and HC samples (Fig. 4). We found that hierarchical clustering did not distinguish samples between GBS versus HC samples or among GBS subtypes, although the dendrogram showed the groups of glycolipid and/or glycolipid complex antibodies may be composed of clusters (Fig. 4).

### EFA of IgG glycoarray data determines five common factors, each of which contains a distinct set of glycolipid antibodies

**Figure 5:**
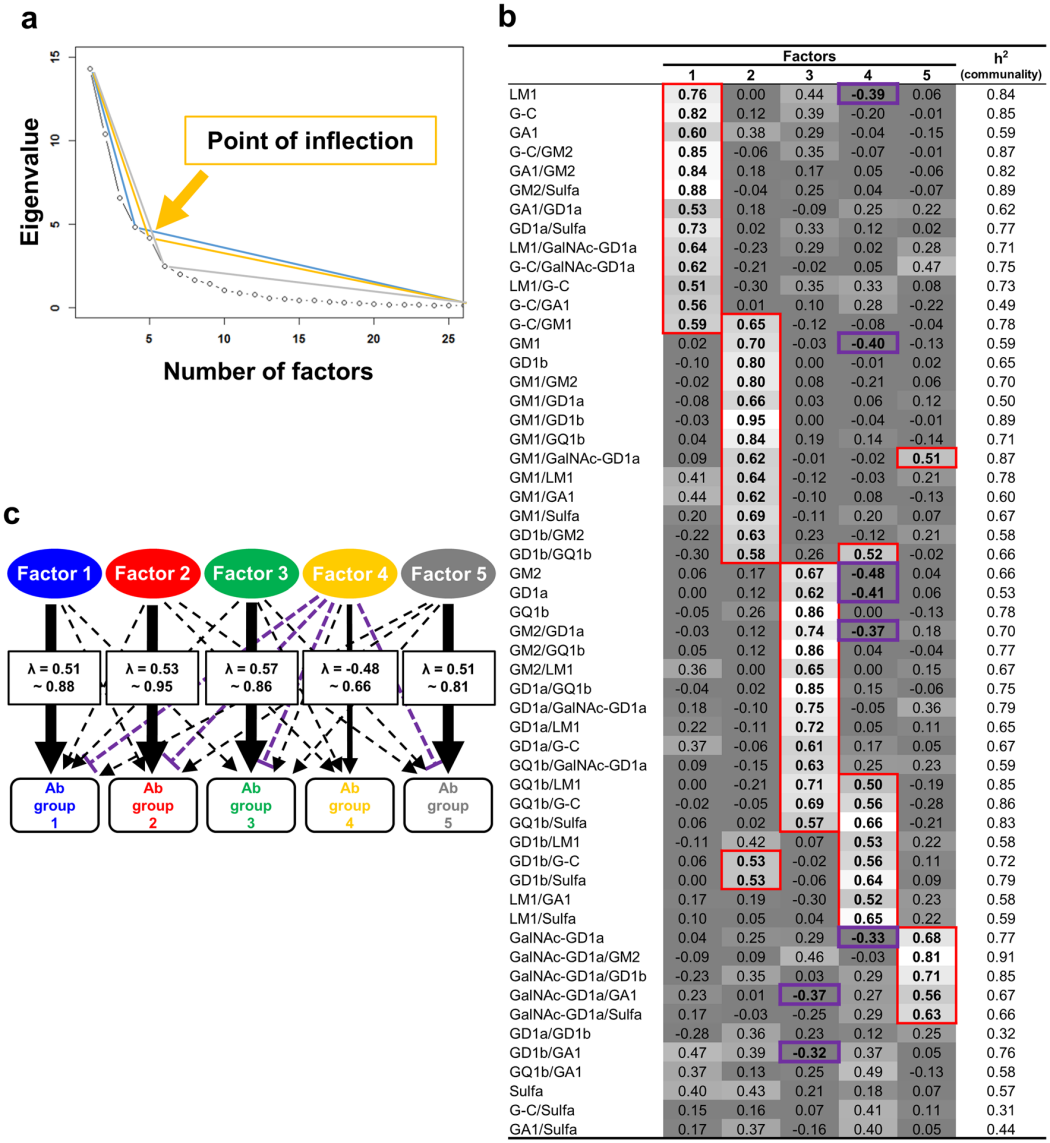
Exploratory factor analysis (EFA) of IgG glycoarray data from GBS patients. (**a**) The point of inflection of the scree plot with the eigenvalues and number of factors indicated that four, five, or six factors were the adequate number of common factors. (**b**) Factor loadings higher than 0.5 (shown in red boxes) were selected as related variables forming the particular factors. We identified five factors: Factor 1 had high factor loadings (≥ 0.5) in LM1, G-C, asialo-GM1 (GA1), and their complexes. Factor 2 had high factor loadings in GM1, GD1b, and their complexes. Factor 3 had high factor loadings in GM2, GD1a, GQ1b, and their complexes. Factor 4 had high factor loadings in GD1b complexes, GQ1b complexes, and LM1 complexes. Factor 4 also had moderately negative factor loadings in LM1, GM1, GM2, GD1a, GM2/GD1a, and GalNAc-GD1a (shown in purple boxes). Factor 5 had high factor loadings of GalNAc-GD1a and their complexes. Six glycolipid antibodies were not categorized into any factors; this could occur when a variable (i.e., glycolipid antibody) was not associated with other variables. (**c**) A model of associations between Factors 1 to 5 with glycolipid antibody (Ab) groups 1 to 5. Each factor was associated with antibodies belonged to distinct Ab group strongly (λ ≥ 0.5) and some antibodies belonged to the other groups moderately (λ ≥ 0.3). Factor 4 was negatively associated with several glycolipid antibodies moderately. A parameter, h^2^, indicates communality that is the ratio (0 to 1) of variance explained by the five Factors.

Since hierarchical clustering of IgG glycoarray data showed possible clustering of several (four to seven) groups of glycolipid and glycolipid complex antibodies, we hypothesized that glycolipids and glycolipid complexes in each cluster might share one common (latent) factor. For example, sensitization with one pathogen containing one common antigen may result in the generation of antibodies to glycolipids and glycolipids complexes that share the common antigen. Since EFA is a statistical method to identify the underlying relationships between measured variables (i.e., glycolipid antibody titers), we conducted EFA, using glycoarray data. We used the scree test to determine the number of common factors: in a scree plot, the number of factors to be retained was the data points that were above the break (i.e., point of inflection)^14^. The scree test indicated that four, five, or six factors were adequate to account for the data (Fig. 5a).

To further determine the most appropriate number of factors, we calculated factor loadings (Fig. 5b, Tables S4, S5, and S6). Factor loadings are measures of the influence of a common factor on a manifest variable (i.e., antibody titers); factor loadings indicate the degree of association between items (i.e., glycolipid antibody titers) and factors (Factors 1 to 4–6). Factor loadings greater than 0.5 were selected as related variables forming the particular factors^15–17^. When we visually compared the three solutions, in the four-factor solution (Table S4), Factor 4 contained four items (antibodies), all of which were also contained in Factor 3 except for LM1/Sulfa. In the six-factor solution (Table S6), Factor 5 contained only two antibodies, GQ1b/G-C and GQ1b/Sulfa, both of whose factor loadings were lower than those of Factor 2 (Factor loadings: GQ1b/G-C, Factor 2 = 0.84, Factor 5 = 0.56; and GQ1b/Sulfa, Factor 2 = 0.71, Factor 5 = 0.64). In the five-factor solution (Fig. 5b), the number of unclassified antibodies into any factors (n = 6) was smaller than the four-factor (n = 8) and six-factor (n = 9) solutions^18^.

Accordingly, we chose the five-factor solution as the best representation of the data; EFA extracted five factors with a more than 8% contribution ratio, whose cumulative contribution ratio of the five factors was 69%^8^. Factor 1 (16% contribution ratio, total 13 antibodies) contained antibodies against LM1, G-C, GA1, and their eight complexes as well as GM2/Sulfa and GD1a/Sulfa (Ab group 1 in Fig. 5c). Factor 2 (17% contribution ratio, total 15 antibodies) contained antibodies against GM1, GD1b, all nine GM1 complexes, and five GD1b complexes, whose common molecule is GM1 (Ab group 2). Factor 3 (17% contribution ratio, total 14 antibodies) contained antibodies against GM2, GD1a, GQ1b, and their 11 complexes, whose common molecule is GM2 (Ab group 3). Factor 4 (11% contribution ratio, total nine antibodies) contained antibodies against four GQ1b complexes, four GD1b complexes, LM1/GA1, and LM1/Sulfa (Ab group 4). Unlike the other Factors, factor loadings of Factor 4 of several glycolipid antibodies showed several negative correlations: LM1 (–0.39), GM1 (–0.4), GM2 (–0.48), GD1a (–0.41), GM2/GD1a (–0.37), and GalNAc-GD1a (–0.33). Factor 5 (8% contribution ratio, total six antibodies) contained antibodies against GalNAc-GD1a and five GalNAc-GD1a complexes (Ab group 5).

### Individual GBS patients have a distinct set of factor scores, which may be associated with clinical signs/symptoms

We calculated the factor scores of individual samples (Table S3). Then, we examined whether each Factor could be associated with distinct clinical signs/symptoms (Table S7). Using a receiver operating characteristic (ROC) analysis, we evaluated whether each Factor could be a biomarker for the following clinical data: sex; antecedent respiratory or digestive infections; involvement of cranial nerves III, IV, V, VI, VII, IX, X, XI, or XII; motor paralysis; respiratory muscle paralysis (with intubation or ventilation); superficial and deep sensory disturbance; ataxia; autonomic nerve involvement; intravenous immunoglobulin (IVIG) treatment; and GBS subtypes including AIDP and AMAN. Among the clinical data, only cranial nerve involvement had an area under the curve (AUC) > 0.7 among all five Factors (Factor 3: cranial nerve IV = 0.72, cranial nerve VI = 0.74; and Factor 4: cranial nerve IV = 0.7, cranial nerve XI = 0.72, Table S7).

We found that each patient seemed to have a unique pattern of the five factor scores. For example, some patients, such as GBS-66 and -69, had low scores in all Factors; GBS-59 and -90 had the highest score in Factor 5; and GBS-100 had a high score only in Factor 2. Thus, we tested whether a group (cluster) of patients could have a unique pattern of factor scores, using *k*-means clustering^19^. Since Davies-Bouldin index showed that the most appropriate cluster number was six (Figure S1b), we separated GBS samples into six clusters, each of which had a unique set of factor scores (Figure S1c and Table S3): Cluster 1, low scores in all Factors; Cluster 2, high score in Factors 5; Cluster 3, high score in Factor 2; Cluster 4, low scores in Factors 2 and 3; Cluster 5, low score in Factor 4; and Cluster 6, high scores in Factors 3 and 4.

We examined whether each Cluster of patients had distinct clinical signs/symptoms (Table S3). Cluster 1 with low scores in all Factors had facial nerve paralysis in 6 of 8 patients (75%, *P* < 0.05 compared with the other five clusters by Pearson’s χ^2^ test) and 6 of 8 patients were equivocal in Hadden’s criteria (75%, *P* < 0.05). Cluster 2 with high GalNAc-GD1a-related antibody titers had more pure motor GBS in 6 of 16 patients (37.5%, *P* < 0.01). Cluster 3 with high titers of GM1- and GD1b-related antibodies had no cranial nerve paralysis in 8 of 11 patients (72.7%, *P* < 0.05) and no respiratory muscle paralysis in all patients (*P* < 0.01). Cluster 4 with low scores in Factors 2 and 3 had sensory abnormalities in 17 of 20 patients (85%, *P* = 0.21) and AIDP in 11 of 20 patients (55%, *P* = 0.06). Cluster 5 with low score in Factor 4 also had sensory abnormalities in 29 of 35 patients (83%, *P* = 0.14). Cluster 6 with high antibodies against GM2, GD1a, GQ1b, and GD1b complexes had paralysis of eye movements in 7 of 10 patients (70%, *P* < 0.01), and 7 of 10 patients were equivocal in Hadden’s criteria (70%, *P* < 0.05). The number of patients whose disease peak days were less than 7 days was 8 of 9 patients (89%) in Cluster 6 and 41 of 77 (53%) in the other GBS patients (*P* < 0.05).

### EFA of IgM glycoarray data extracts the different number and nature of factors, compared with those of IgG

We tested whether bioinformatics analysis of IgM glycoarray data (Table S2) could be useful for the diagnosis or prognosis of GBS subtypes. First, we conducted PCA, using glycolipid and glycolipid complex IgM antibody titers of GBS and HC samples (Fig. 3b). Although some GBS samples have high PC1 and/or PC2 values, PCA did not separate distinct populations among the GBS subtypes and HC samples.

**Figure 6:**
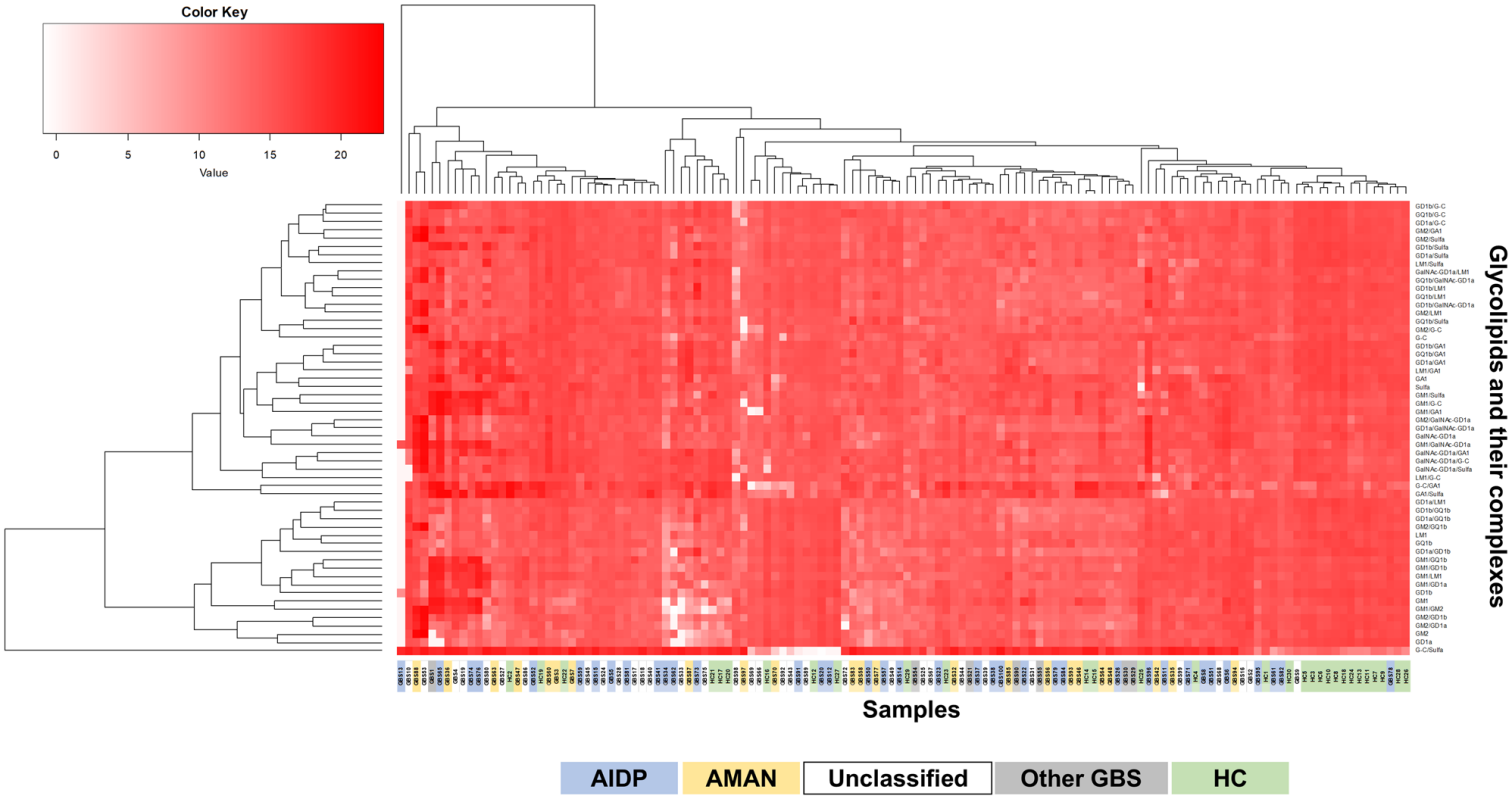
A heat map of serum anti-glycolipid IgM antibody titers in GBS subtypes and HC samples. We quantified 10 anti-glycolipid and 45 anti-glycolipid complex antibodies by IgM glycoarray and aligned individual samples, using hierarchical clustering. Each row represents anti-glycolipid antibody titers against a single glycolipid antigen, and each column represents a single sample. Red density indicates the antibody titer level; white indicates the absence of the antibody. A dendrogram at the top is the hierarchical clustering based on the IgM titer pattern of individual samples. Color bars at the bottom of the heat map indicate that the samples from AIDP, blue; AMAN, orange; “Unclassified," white; “Other GBS”, gray; and HC, green, showing the dispersal of GBS subgroups and HC samples. A dendrogram at the top of the heat map did not separate the GBS subtypes and HC samples clearly. In the dendrogram aligned based on glycolipid and glycolipid complex antigens (at the left of the heat map), GalNAc-GD1a and six GalNAc-GD1a complex IgM antibodies were clustered at the middle of the dendrogram. GM1 and GM1 complex IgM antibodies seemed to belong to, at least, two different clusters.

Next, we drew a heat map with dendrograms based on IgM glycolipid and glycolipid complex antibody titers of all GBS and HC samples (Fig. 6). A dendrogram at the top of the heat map did not separate GBS subtypes and HC samples clearly, although some small clusters were mostly composed of the same GBS subtypes or HC (e.g., two small clusters at the right side of the heat map contained 13 HC samples among the total 15 samples). The dendrogram aligned based on glycolipid and glycolipid complex antigens (at the left of the heat map) seemed to reflect different clustering from that of IgG anti-glycolipid antibodies. For example, we found that GalNAc-GD1a and six GalNAc-GD1a complex IgM antibodies were clustered in the middle of the dendrogram and that GM1 and GM1 complex IgM antibodies seemed to belong to, at least, two different clusters (Fig. 6).

**Figure 7:**
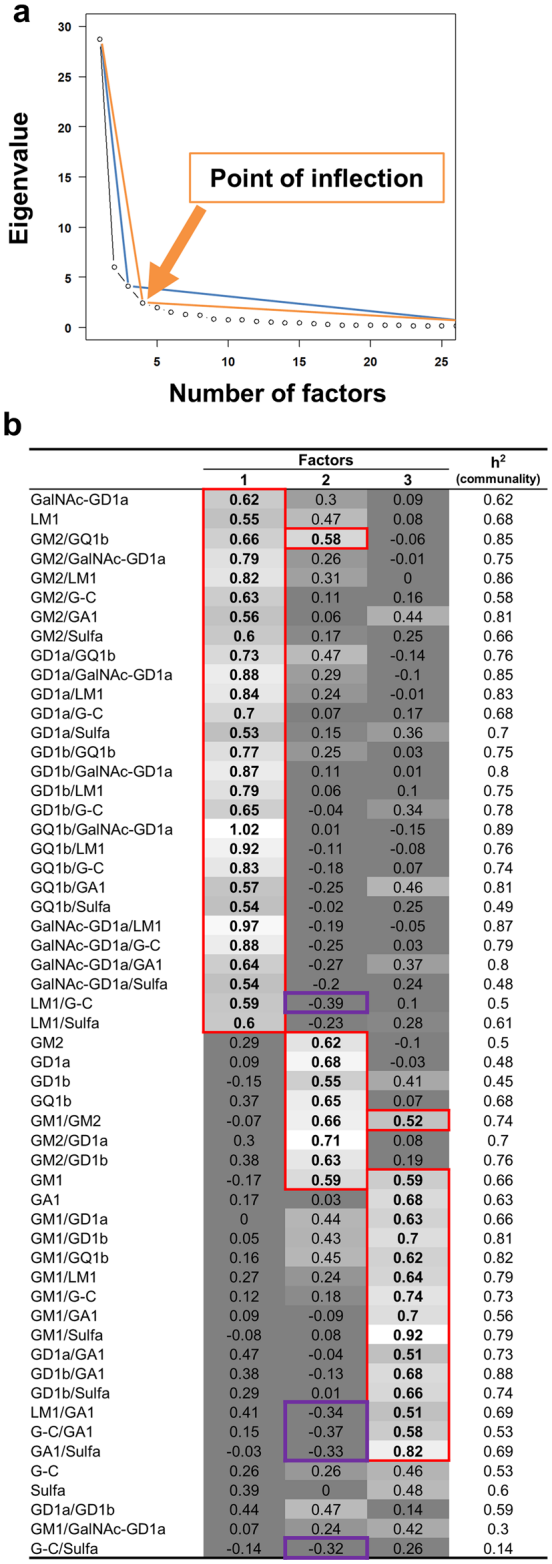
EFA of IgM glycoarray data from GBS patients. (**a**) The point of inflection of the scree plot with the eigenvalues and number of factors indicated that three or four factors were the adequate number of common factors. (**b**) Factor loadings greater than 0.5 (shown in red boxes) or less than –0.3 (shown in purple boxes) were selected as related variables forming the particular factors. We identified three common factors: Factor 1 had high factor loadings in GalNAc-GD1a, LM1, and glycolipid complexes, including GalNAc-GD1a, LM1, G-C, GA1, Sulfa, and GQ1b. Factor 2 had high factor loadings in GM1, GM2, GD1a, GD1b, GQ1b, and their complexes. Factor 2 also had moderately negative factor loadings in LM1/G-C, LM1/GA1, G-C/GA1, GA1/Sulfa, and G-C/Sulfa. Factor 3 had high factor loadings in GM1, GA1, and their complexes. A parameter, h^2^, indicates communality that is the ratio (0 to 1) of variance explained by the three Factors.

We conducted EFA, using IgM glycolipid antibody titers of GBS patients (Fig. 7a,b, and Tables S8, S9). The scree test indicated that three or four factors were adequate to account for the data. In visually comparing the two solutions, we found that the three-factor solution contained fewer antibodies whose factor loadings were high in more than one factor, and that the number of unclassified antibodies into any factors (n = 5) (Fig. 7b and Table S8) was smaller than that of the four-factor solution (n = 6) (Table S9). Thus, we chose the three-factor solution, whose cumulative contribution ratio of the three factors was 69%. Factor loadings for each factor showed that Factors 1 to 3 of IgM glycoarray contained different sets of glycolipid and glycolipid complex antibodies (Fig. 7b), compared with those of IgG glycoarray (Fig. 5b). For example, among GM1 and GM1 complex antibodies, IgM factor loading of GM1 and GM1/GM2 were high in two factors (GM1, Factor 2 = 0.59, Factor 3 = 0.59; and GM1/GM2, Factor 2 = 0.66, Factor 3 = 0.52); IgG factor loadings of GM1/GalNAc-GD1a and GM1/G-C were high in different two factors (GM1/GalNAc-GD1a, Factor 2 = 0.62, Factor 5 = 0.51; and GM1/G-C, Factor 1 = 0.59, Factor 2 = 0.65). In Factor 2, factor loadings of several glycolipid complex IgM antibodies showed negative correlations: LM1/G-C (–0.39), LM1/GA1 (–0.34), G-C/GA1 (–0.37), and G-C/Sulfa (–0.32). Thus, the number of common factors and their clustering pattern of IgM glycolipid antibodies were different from those of IgG; this may reflect that the presence of distinct latent factors to induce IgM versus IgG antibody production, for example, different antigen recognition by IgM and IgG.

We calculated the IgM factor scores of individual samples (Table S10). Using an ROC analysis, we found no association between the three IgM factors and clinical signs/symptoms (Table S11). Lastly, although we separated GBS patients into four clusters by k-means clustering of factor scores, no cluster of patients had distinct clinical signs/symptoms, compared with patients belonged to the other clusters (Table S10).

## Discussion

It has not been explored how many major triggers are present or how many major PNS structures can be autoantigens in GBS. This can be due to a large number of candidates of triggers and autoantigens that have been demonstrated in GBS research. Although a variety of clinical signs/symptoms have been observed in GBS, it is reasonable to hypothesize that a limited number of triggers/autoantigens are responsible for the pathogenesis of most GBS cases, whose subgroups share clinical and electrophysiological features. In this manuscript, we demonstrated that EFA enables us to determine the number and the nature of common latent factors underlying the production of 55 glycolipid antibodies, using glycoarray data.

**Figure 8:**
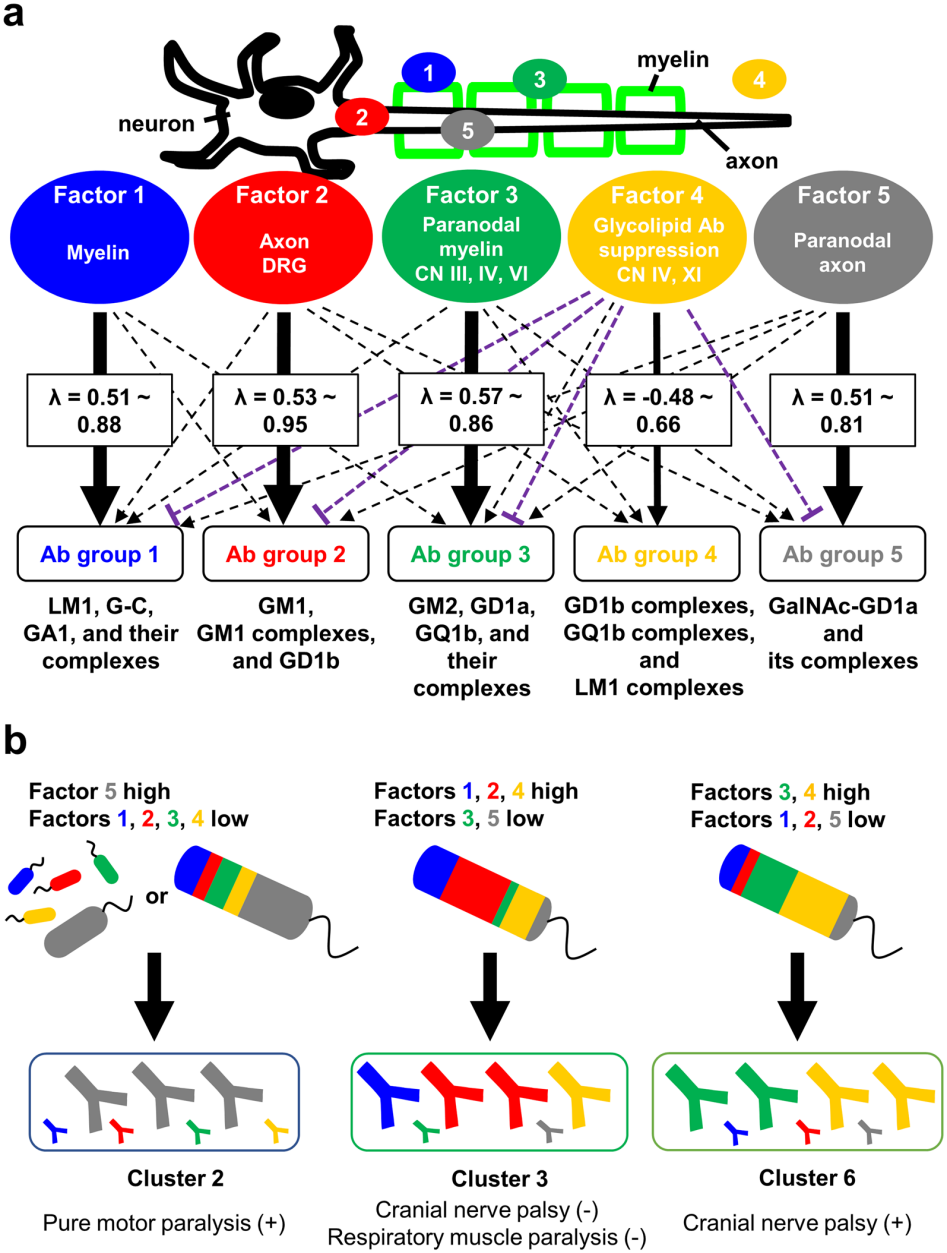
Working models of how each factor associates a set of glycolipid antibodies. (**a**) Based on the factor loadings, each factor was associated with a distinct set of anti-glycolipid antibodies (Ab). Each factor, except for Factor 4, was associated with distinct glycolipid antigens in the peripheral nervous system. Factor 1 was associated strongly with antibodies against LM1, G-C, GA1, and their complexes, Factor 1 can be myelin antigens. Factor 2 contains antibodies against GM1 and GM1 complexes, which are components of the axonal membrane, and GD1b, whose antibody can bind the dorsal root ganglia (DRG). Factor 3 contains the antibodies against GM2, GD1a, and GQ1b, which are paranodal myelin antigens of cranial nerves (CN) III, IV, and VI. Factor 4, which was associated with involvement of CN IV and XI, may be a latent factor that suppresses the production of a group of anti-glycolipid antibodies. Factor 5 contains the antibodies against GalNAc-GD1a and its complexes, which are components of the paranodal axonal membrane. (**b**) GBS patients could be classified into six clusters based on *k*-means clustering of our EFA results. Each cluster has a unique pattern of five factors (Factor 1 to Factor 5). For example, sera of patients in Cluster 2 had high factor scores in Factor 5. Sera of patients in Cluster 3 had high factor scores in Factors 1, 2, and 4. Sera of patients in Cluster 6 had high factor scores in Factors 3 and 4. In theory, the difference in the factor score patterns among six clusters can be due to simultaneous multiple exposures by several factors, each of which have a different antigenicity level (upper left of Cluster 2 figure), or an infection with one microbe containing multiple antigens with different antigenicities (upper right of Cluster 2 figure, and Cluster 3 and Cluster 6 figures), leading to the production of unique sets of glycolipid antibodies or causing specific clinical signs/symptoms.

Using IgG glycoarray of GBS sera, we identified five factors (Fig. 5b), which can be associated with the presence of glycolipid antigens on the PNS, except for Factor 4 (Fig. 8a). Factor 1 contains antibodies against LM1, G-C, and GA1, which are components of the myelin sheaths. We did not see a strong association between Factor 1 and clinical signs/symptoms. Factor 2 contains antibodies against GM1 and all nine GM1 complexes, which are components of the axonal membrane. Factor 2 also contains GD1b and two of their complexes, GD1b/GM1 and GD1b/GM2, but not the other GD1b complexes; this is consistent with our previous finding that monospecific GD1b antibody did not react with most GD1b complexes, but a few of them including GD1b/GM1^20^. GD1b antibody has been shown to bind the dorsal root ganglia (DRG), experimentally^21^. Factor 3 contains the antibodies against GM2, GD1a, and GQ1b, which are paranodal myelin antigens of cranial nerves III, IV, and VI^22–25^. This is consistent with a report of the association between anti-GQ1b antibody and ophthalmoplegia^25^ as well as our current ROC analysis between Factor 3 and cranial nerve involvement (AUC: III, 0.69; IV, 0.72; and VI, 0.74, Table S7). Factor 5 contains the antibodies against GalNAc-GD1a and their complexes, which are components of the paranodal axonal membrane. Since Kaida et al.^26^ reported the association between anti-GM1/GalNAc-GD1a complex antibody and pure motor GBS, we conducted ROC analysis between Factor 5 and pure motor GBS, and found acceptable discrimination of Factor 5 (with respiratory antecedent infection, AUC = 0.76; without respiratory antecedent infection, AUC = 0.64).

Factor 4 contains the antibodies against nine glycolipid complex antibodies composed of LM1, G-C, Sulfa, GQ1b, or GD1b. AUC of our ROC analysis showed the association between Factor 4 and involvement of cranial nerves IV and XI. The association with cranial nerve IV involvement is consistent with previous findings of serum anti-GQ1b complex antibodies from patients with ophthalmoplegia^7^. On the other hand, no anti-glycolipid antibody has been reported in patients with cranial nerve XI involvement. Although our current EFA was based on highly positive factor loadings of glycolipid antibodies, only Factor 4 had several negative correlations with several monospecific glycolipid antibodies (factor loadings: LM1, –0.39; GM1, –0.40; GM2, –0.48; GD1a, –0.41). Thus, Factor 4 might be a latent factor that suppresses particular anti-glycolipid antibody production. The presence of a similar potential suppressive latent factor was also suggested in EFA of IgM glycoarray data, in which we found the negative correlations between IgM Factor 2 and antibodies against glycolipid complexes composed of LM1, G-C, GA1, and Sulfa (Fig. 7b).

In IgM glycolipid EFA, we found that the number of Factors and their clustering were different from those of IgG (Figs. 5b and 7b, Tables S5 and S8). This is consistent with previous findings that IgM glycolipid antibody titers and patterns and those of IgG in GBS were often different^27^. Although there is no clear explanation for this discrepancy between IgM and IgG anti-glycolipid antibody production, our current EFA results suggest that the presence of distinct latent factors to induce IgM versus IgG antibody production. Currently, although we do not know what latent factors affect IgM versus IgG glycolipid antibody productions, the candidate factors include different causative antigens for IgM versus IgG, class-switching cytokines favoring either isotype, and production of natural IgM antibodies with polyreactivity.

It is unclear what latent factor can inhibit the production of a group of IgG glycolipid antibodies or IgM glycolipid complex antibodies. Inhibition of the reactivities of some glycolipid antibodies by other coexistent glycolipids have been observed in vitro^20,22^; this is due to the interaction of two glycolipids that creates a new epitope with conformational changes, leading to the discovery of antibodies against glycolipid complexes. Sphingomyelin may also affect glycolipid epitopes; in anti-GM1 and GQ1b antibody ELISA, anti-GM1 and anti-GQ1b antibody activities have been shown to decrease when a mixture of sphingomyelin and GM1 or GQ1b was coated on the ELISA plate, compared with the plates coated with GM1 or GQ1b alone^28^. Similar changes might be induced in vivo by as yet unknown IgG Factor 4 or IgM Factor 2; for example, since free glycolipids can be released following the damage of the nervous system^29^, these glycolipids might bind to distinct glycolipid epitopes on nerve structures, reducing their antigenicity. Alternatively, several factors, including infections^30,31^, diet^29^, drugs^32^, and aging^33^, have been demonstrated to alter the expression of a group of glycolipid expressions in the PNS.

Clinical signs/symptoms by one glycolipid antibody may depend on the presence^34,35^ or absence of other glycolipid antibodies in one GBS patient. For example, although GBS patients with anti-G-C antibodies tended to have demyelinating neuropathy, Susuki et al.^36^ showed that GBS patients with anti-G-C and GM1 antibodies exhibiting axonal neuropathy. In contrast, Samukawa et al.^37^ showed that GBS patients with both G-C and ganglioside antibodies including GM1 were categorized as AIDP. The discrepancy between these results suggests that one glycolipid antibody response may not be sufficient to cause (or result from) a specific type of neuronal damage. Thus, there is a gap of knowledge as to whether one glycolipid antibody or a combination of these antibodies is pathogenic (or biomarker) of GBS subtypes. Our EFA of glycoarray is the first comprehensive attempt to address the question by conducting *k*-means clustering to classify GBS samples into five clusters. The clustering was based on the patterns of the five factor scores, each of which reflected the levels of a set of glycolipid antibodies that were components of one of five Factors. The distinct pattern based on the levels of groups of antibodies can be due to the presence of different amounts of latent factors, e.g., one bacterial infection with four minor factors (other environmental factors, sex, genetics, etc.). Alternatively, this can be due to the presence of one latent factor that can induce five different outcomes, e.g., infection with one microbe whose cell membrane contains various levels of distinct glycolipids-like antigens (Fig. 8b).

In this scenario, we anticipated that a group of glycolipid antigens belonging to one factor or one cluster could be explained by antecedent infections by certain microbes, such as *Campylobacter jejuni* and Japanese encephalitis virus^38^. Among 100 GBS patients of this study, 70 patients had antecedent infections (respiratory, 43; digestive, 23; both, 4) (Table S3). Although the incidence of antecedent infections was significantly high among GBS patients (*P* < 0.01 by χ^2^ test), neither respiratory nor digestive antecedent infections were significantly associated with Factors or Clusters (Tables S7 and S11). Among 70 antecedent infections in this study, microbes were identified only in three cases (two mycoplasma and one influenza A virus); we were not able to associate any microbial infections with anti-glycolipid antibody productions.

Initially, we anticipated that, once latent factors were determined (i.e., Factor association with myelin antigen, Factor 2 association with axonal/DRG antigen), each factor should be associated with major clinical signs or GBS subtypes including AIDP and AMAN. However, this was not the case. The lack of association between factor scores and GBS subtypes could be due to the inclusion of glycoarray data of “Other GBS”. Thus, we performed a hierarchical clustering without “Other GBS” data and found that the clustering did not distinguish samples between GBS subtypes and HC (Figure S2). We also conducted EFA without “Other GBS” data and found that the number of factors and their containing antibodies based on the EFA without “Other GBS” data were similar to the EFA with “Other GBS” data (Table S12).

The lack of association was most likely because each patient had a unique pattern of the five factor scores; clinical signs/phenotypes could depend on the presence or absence of other factors. Therefore, we conducted *k*-means clustering, using factor scores (Figures S1c and S1e). Using IgG data, we separated GBS samples into six clusters, each of which had a unique set of factor scores (Figure S1c). We attempted to characterize Clusters 1 to 6 with distinct clinical signs/symptoms, although not all Clusters can be distinguished by clinical data available in our data set (Table S3). For example, all patients in Cluster 2 with high scores in Factor 5 containing GalNAc-GD1a-related antibodies had more pure motor GBS patients. Cluster 3 with high scores in Factors 1, 2, and 4, containing GM1- and GD1b-related antibodies, had no cranial nerve paralysis in 8 of 11 patients and no respiratory muscle paralysis in all patients. Cluster 6 with high scores in Factors 3 and 4, containing antibodies against GM2, GD1a, GQ1b, and GD1b complexes, had cranial nerve involvement in 7 of 10 patients and early disease peak (< 7 days) in 8 of 9 patients. On the other hand, using IgM data, although we separated the samples into four clusters (Figure S1e), we were not able to find any associations between the clusters and clinical data (Table S10).

In our current EFA, there were several limitations, for example, we were not able to find an association between IgM Factors with neuroantigens or clinical signs/symptoms. This could be addressed by an increase in the number of the GBS samples; optimization of antigen densities on glycoarray; exclusion of highly mono-specific antibody associated with unique symptomatology, such as GD1a/GD1b antibody^39^, from EFA; and inclusion of other data, particularly information about the antecedent infections with specific pathogens.

Lastly, we believe that by optimizing experimental conditions of glycoarrays, we will be able to conduct a confirmatory factor analysis, which can be used to determine the diagnosis and prognosis of GBS patients, and that the application of EFA in other biomedical big data should provide insights into the pathomechanisms of diseases with unknown causes and/or risk factors.

In conclusion, using EFA of anti-glycolipid antibody data, we were able to determine five latent factors in GBS. Four factors were related to anatomically distinct neuroantigens: each factor contained a set of anti-glycolipid antibodies against surface antigens on either 1) myelin, 2) axon/dorsal root ganglion, 3) paranodal myelin, or 4) paranodal axon. Here, unsupervised EFA of anti-glycolipid antibody data was able to classify the antibodies into distinct groups of anti-neuronal antibodies. The classification was consistent with experimental and clinical findings of anti-glycolipid antibodies that have been shown to bind specific neuronal structures. Furthermore, EFA extracted one potentially suppressive factor, which has not been reported previously. Therefore, using anti-glycolipid antibodies data of GBS patients as a model system, we demonstrated that EFA could be applicable to biomedical data to extract latent factors. Applying EFA for other biomedical big data may also elucidate latent factors of other diseases with unknown causes or suppressing/exacerbating factors, including coronavirus disease 2019 (COVID-19).

## Methods

### Serum samples

We obtained sera from 100 patients with GBS [44 males and 56 females, median age 54.4 (20–91 years old), Table S3] at Kindai University Hospital (Osakasayama, Osaka, Japan) and other institutes in Japan, as we described previously^1^. HC sera were obtained from 30 healthy volunteers with no microbial infections (20 males and 10 females, from the late 20s to late 40s) at Kindai University Hospital. We diagnosed GBS patients using criteria established by Asbury and Cornblath^11^, and classified the patients electrophysiologically using criteria established by Ho et al.^12^ into three groups: AIDP, AMAN, and “Unclassified” (Table S3), or using criteria established by Hadden et al. into five groups: normal (N), primary demyelinating (D), primary axonal (A), inexcitable (I), and equivocal (E)^13^. GBS patients with few or no electrophysiological data were classified as “Other GBS”. The serum samples from patients with Miller Fisher syndrome or Bickerstaff brainstem encephalitis were not included in our current study. This study was approved by the Internal Review Board of the Kindai University Faculty of Medicine (Osakasayama, Osaka, Japan).

### Combinatorial glycoarray analysis

We conducted a combinatorial glycoarray, as we described previously^1^. We diluted stocked glycolipid solutions of GM1 (G7641, SIGMA, Saint Louis, MO), GM2 (G8397, SIGMA), GD1a (G2392, SIGMA), GD1b (G8146, SIGMA), GQ1b (345,754, CALBIOCHEM, San Diego, CA), GalNAc-GD1a (8G16-17b, HyTest, Turku, Finland), LM1 (provided by Dr. Masao Iwamori, Kindai University, Osaka, Japan), galactocerebroside (G-C) (C4905, SIGMA), asialo-GM1 (GA1) (G3018, SIGMA) and sulfatide (Sulfa) (S1006, SIGMA) to 100 μg/ml with methanol and made the glycolipid complexes by mixing equal volumes of the two different glycolipid solutions. We detected serum antibodies against 10 glycolipids and 45 glycolipid complexes spotted onto Immobilon-FL polyvinylidene difluoride (PVDF) membranes (Millipore Sigma, Burlington, MA) that were affixed to glass slides. We blocked the PVDF membranes with 2% bovine serum albumin (BSA) in phosphate-buffered saline (PBS) for 1 hour (h) at room temperature, diluted serum samples at 1:100 with 1% BSA in PBS, and applied them to the PVDF membrane on glass slides for 2 h at 4°C. After washing three times with 0.1% BSA in PBS, we applied Alexa 647 anti-human IgM and Alexa 555 anti-human IgG antibodies (Thermo Fisher Scientific, Waltham, MA, USA, 1:1000 dilution with 0.1% BSA in PBS) as secondary antibodies for 1 h, washed with 0.1% BSA in PBS as well as distilled water, and quantified fluorescent intensities by Image Quant TL software (GE Healthcare, Chicago, IL). Previously, we evaluated the glycoarray data based on the seropositivity of anti-glycolipid antibodies, comparing with the mean fluorescent intensities of control samples^1^. In this study, instead of seropositivity, we used the absolute value of fluorescent intensities of glycolipid antibody titers (Tables S1 and S2), which were converted to the binary logarithm for PCA and EFA.

### Bioinformatics analyses

#### Principal component analysis (PCA)

Using PCA, we reduced the dimensionality of a glycoarray data set consisting of 55 antibody titers into two components, PC1 and PC2. We conducted PCA to visualize the variance among glycolipid antibody titers of GBS and HC samples, using R version 3.6.0 and an R function ‘prcomp’, as we described previously^40^. We ran a ‘prcomp’ program using the binary-logarithmized data of glycolipid antibody titers and drew a graph of the sample distribution on PC1 and PC2 axes. An ellipse of the 99% confidence interval was drawn, using R packages “dplyr” and “ggplot2”. We calculated the proportion of variance to determine the percentage of variance explained by each PC, as well as factor loading for PC1 to see the correlation between anti-glycolipid antibody titers and PC1 value.

#### Heat map

We drew a heat map with dendrograms to classify the samples based on the patterns of glycolipid antibody titers. We ran the R packages, ‘gplots’ and ‘genefilter’ using binary-logarithmized data of glycolipid antibody titers^19^. Hierarchical clustering was conducted and shown as dendrograms.

#### Exploratory factor analysis (EFA)

We conducted EFA of glycoarray data, using an R package “Psych”^41,42^. In EFA, there are several methods for factor extraction and rotation, which have been improved over the years^43^. A maximum-likelihood method for factor extraction and a geominQ rotation for factor rotation have been highly evaluated among the methods^44,45^, regardless of which statistical software is used. A maximum-likelihood method is most widely used; a geominQ rotation is useful for data with complex structure. We used the scree test to determine the most appropriate number of factors to retain^46^. The scree plot is a graph of the reduced correlation matrix’s eigenvalues plotted in descending order; the last sharp drop in values ("point of inflection") represents the number of factors to be retained. We calculated factor loading for each antibody and factor score for each patient. Factor loadings indicate the degree of association between each glycolipid antibody titers and factors. Factor loadings = 0.5 is frequently used to cut-off between high and low factor loadings^17^, and Comrey and Lee (1992)^16^ suggested to use cut-offs going from 0.32 (poor), 0.45 (fair), 0.55 (good), 0.63 (very good), or 0.71 (excellent). We defined that factor loadings whose values were higher than 0.5 as a “good” association to configure the factors^15^.

In EFA, a popular recommendation regarding the minimum necessary sample size is n = 100^47^. EFA can be reliable for sample sizes well below 50^48^, if communalities are high^47,49^. We confirmed that communalities (= h^2^) were sufficiently high in all EFA (Figs. 5b and 7b, Tables S4, S5, S6, S8, and S9), in which most h^2^ exceeded 0.5. Although we found that several anti-glycolipid antibody titers did not show a normal distribution by Kolmogorov–Smirnov test, a maximum-likelihood method based on the covariance matrix has been widely used in empirical studies due to its robustness to deviations from a multivariate normal distribution^50^.

#### K-means clustering

We conducted *k*-means clustering to cluster the patients based on the patterns of factor scores, using an R package ‘cclust’^19^. Davies-Bouldin index was used to determine the optimum number of clusters. A radar chart of cluster centers was drawn, using OriginPro (OriginLab, Northampton, MA).

#### Receiver operating characteristic (ROC) analysis

We conducted an ROC analysis to evaluate the association between the factor scores and clinical data, using an R package "ROCR". We considered the area under the curve (AUC) of: 0.5, no discrimination; > 0.5, reasonable discrimination; 0.7 to 0.8, acceptable discrimination; 0.8 to 0.9, excellent discrimination; and more than 0.9, outstanding discrimination (i.e., ability to diagnose patients with and without the disease or condition based on the test)^51^.

### Ethics approval

This study was approved by the Internal Review Board of the Kindai University Faculty of Medicine (Osakasayama, Osaka, Japan).

## Supplementary Information

Supplementary Figures.

Supplementary Tables.

## Data Availability

All data generated or analyzed during this study were included in this published article and its supplementary information files (Tables S1-S12).

## References

[1] Morikawa M (2016). Serological study using glycoarray for detecting antibodies to glycolipids and glycolipid complexes in immune-mediated neuropathies. J. Neuroimmunol..

[2] Tsunoda I, Fujinami RS (2002). Inside-Out versus Outside-In models for virus induced demyelination: Axonal damage triggering demyelination. Springer Semin. Immunopathol..

[3] Kaida K, Kusunoki S, Kamakura K, Motoyoshi K, Kanazawa I (2003). GalNAc-GD1a in human peripheral nerve: target sites of anti-ganglioside antibody. Neurology.

[4] Tsunoda I (2016). Neuropathogenesis of Zika virus infection: potential roles of antibody-mediated pathology. Acta Med. Kinki Univ..

[5] Dutta D (2021). Antecedent infections in Guillain–Barré syndrome patients from south India. J. Peripher. Nerv. Syst. JPNS.

[6] Rinaldi S, Brennan KM, Willison HJ (2012). Combinatorial glycoarray. Methods Mol. Biol..

[7] Halstead SK (2016). Microarray screening of Guillain–Barré syndrome sera for antibodies to glycolipid complexes. Neurol. Neuroimmunol. Neuroinflammation.

[8] Mooi, E., Sarstedt, M. & Mooi-Reci, I. Principal component and factor analysis. in *Market Research* 265–311 (Springer Singapore, 2018). 10.1007/978-981-10-5218-7_8.

[9] Fabrigar LR, Wegener DT, MacCallum RC, Strahan EJ (1999). Evaluating the use of exploratory factor analysis in psychological research. Psychol. Methods.

[10] Chaitanya, G. V. *et al.* Inflammation induces neuro-lymphatic protein expression in multiple sclerosis brain neurovasculature. *J. Neuroinflammation* **10**, 125. 10.1186/1742-2094-10-125 (2013). 10.1186/1742-2094-10-125 PMC3854084 24124909

[11] Asbury AK, Cornblath DR (1990). Assessment of current diagnostic criteria for Guillain–Barré syndrome. Ann. Neurol..

[12] Ho TW (1995). Guillain–Barré syndrome in northern China. Relationship to Campylobacter jejuni infection and anti-glycolipid antibodies. Brain.

[13] Hadden RDM (1998). Electrophysiological classification of Guillain–Barré syndrome: Clinical associations and outcome. Ann. Neurol..

[14] Yong AG, Pearce S (2013). A beginner’s guide to factor analysis: Focusing on exploratory factor analysis. Tutor. Quant. Methods Psychol..

[15] Gorsuch, R. L. *Factor analysis*. (Lawrence Erlbaum Associates, 1983).

[16] Comrey, A. L. & Lee, H. B. *A first course in factor analysis*. (Lawrence Erlbaum Associates, 1992).

[17] Maskey R, Fei J, Nguyen H-O (2018). Use of exploratory factor analysis in maritime research. Asian J. Shipp. Logist..

[18] Shimizu K, Vondracek FW, Schulenberg JE, Hostetler M (1988). The factor structure of the career decision Scale: Similarities across selected studies. J. Vocat. Behav..

[19] Omura S (2014). Bioinformatics multivariate analysis determined a set of phase-specific biomarker candidates in a novel mouse model for viral myocarditis. Circ. Cardiovasc. Genet..

[20] Kaida K (2008). GD1b-specific antibody induces ataxia in Guillain–Barré syndrome. Neurology.

[21] Takada K, Shimizu J, Kusunoki S (2008). Apoptosis of primary sensory neurons in GD1b-induced sensory ataxic neuropathy. Exp. Neurol..

[22] Kusunoki S, Kaida K (2011). Antibodies against ganglioside complexes in Guillain–Barré syndrome and related disorders. J. Neurochem..

[23] Kaida K, Kusunoki S (2010). Antibodies to gangliosides and ganglioside complexes in Guillain–Barré syndrome and Fisher syndrome: Mini-review. J. Neuroimmunol..

[24] Chiba A, Kusunoki S, Obata H, Machinami R, Kanazawa I (1993). Serum anti-GQ_1b_ IgG antibody is associated with ophthalmoplegia in Miller Fisher syndrome and Guillain–Barré syndrome: clinical and immunohistochemical studies. Neurology.

[25] Chiba A, Kusunoki S, Obata H, Machinami R, Kanazawa I (1997). Ganglioside composition of the human cranial nerves, with special reference to pathophysiology of Miller Fisher syndrome. Brain Res..

[26] Kaida K (2008). GM1/GalNAc-GD1a complex: a target for pure motor Guillain–Barre syndrome. Neurology.

[27] Koga M, Takahashi M, Yokoyama K, Kanda T (2015). Ambiguous value of anti-ganglioside IgM autoantibodies in Guillain–Barré syndrome and its variants. J. Neurol..

[28] Hirakawa M, Morita D, Tsuji S, Kusunoki S (2005). Effects of phospholipids on antiganglioside antibody reactivity in GBS. J. Neuroimmunol..

[29] Yaqoob P (2009). The nutritional significance of lipid rafts. Annu. Rev. Nutr..

[30] Hanada K (2005). Shingolipids in infectious disease. Jpn. J. Infect. Dis..

[31] Ang CW (2000). Cross-reactive antibodies against GM2 and CMV-infected fibroblasts in Guillain–Barré syndrome. Neurology.

[32] Aerts, J. M. F. G., Artola, M., van Eijk, M., Ferraz, M. J. & Boot, R. G. Glycosphingolipids and infection. Potential new therapeutic avenues. *Front. Cell Dev. Biol.* **7**, 324. 10.3389/fcell.2019.00324 (2019). 10.3389/fcell.2019.00324 PMC6908816 31867330

[33] Caughlin S (2017). Age-dependent and regional heterogeneity in the long-chain base of A-series gangliosides observed in the rat brain using MALDI Imaging. Sci. Rep..

[34] Kaida K (2007). Anti-ganglioside complex antibodies associated with severe disability in GBS. J. Neuroimmunol..

[35] Ogawa G (2009). Antibodies to ganglioside complexes consisting of asialo-GM1 and GQ1b or GT1a in Fisher and Guillain–Barré syndromes. J. Neuroimmunol..

[36] Susuki K, Odaka M, Mori M, Hirata K, Yuki N (2004). Acute motor axonal neuropathy after *Mycoplasma* infection: Evidence of molecular mimicry. Neurology.

[37] Samukawa M (2016). Electrophysiological assessment of Guillain–Barré syndrome with both Gal-C and ganglioside antibodies; tendency for demyelinating type. J. Neuroimmunol..

[38] Dutta D (2022). Impact of antecedent infections on the antibodies against gangliosides and ganglioside complexes in Guillain–Barré syndrome: A correlative study. Ann. Indian Acad. Neurol..

[39] Hamaguchi T (2007). Guillain–Barré syndrome with antibodies to GD1a/GD1b complex. J. Neurol. Neurosurg. Psychiatry.

[40] Omura, S. *et al.* Bioinformatics analyses determined the distinct CNS and peripheral surrogate biomarker candidates between two mouse models for progressive multiple sclerosis. *Front. Immunol.* **10**, 516. 10.3389/fimmu.2019.00516 (2019). 10.3389/fimmu.2019.00516 PMC6434997 30941144

[41] R Core Team. *R: A Language and Environment for Statistical Computing*. (R Foundation for Statistical Computing, 2018).

[42] Revelle, W. *psych: Procedures for Psychological, Psychometric, and Personality Research*. (Northwestern University, 2018).

[43] Jöreskog KG (1967). Some contributions to maximum likelihood factor analysis. Psychometrika.

[44] Norris M, Lecavalier L (2010). Evaluating the use of exploratory factor analysis in developmental disability psychological research. J. Autism Dev. Disord..

[45] Browne MW (2001). An overview of analytic rotation in exploratory factor analysis. Multivar. Behav. Res..

[46] Cattell RB (1966). The scree test for the number of factors. Multivar. Behav. Res..

[47] Preacher KJ, MacCallum RC (2002). Exploratory factor analysis in behavior genetics research: Factor recovery with small sample sizes. Behav. Genet..

[48] de Winter JCF, Dodou D, Wieringa PA (2009). Exploratory factor analysis with small sample sizes. Multivar. Behav. Res..

[49] MacCallum RC, Widaman KF, Zhang S, Hong S (1999). Sample size in factor analysis. Psychol. Methods.

[50] Benson J, Fleishman JA (1994). The robustness of maximum likelihood and distribution-free estimators to non-normality in confirmatory factor analysis. Qual. Quant..

[51] Mandrekar JN (2010). Receiver operating characteristic curve in diagnostic test assessment. J. Thorac. Oncol..

