# Supplementary material for: Exploratory factor analysis determines latent factors in Guillain–Barré syndrome
Source: Sci Rep. 2022 Dec 17;12:21837. doi: 10.1038/s41598-022-26422-5 (PMC9758666; doi:10.1038/s41598-022-26422-5)
Supplement: Supplementary file 1 — Supplementary Figures. [file 41598_2022_26422_MOESM1_ESM.docx]

**Supplemental Materials**

**Exploratory factor analysis determines latent factors in Guillain-Barré syndrome**

Seiichi Omura^1^, Kazuaki Shimizu^3^, Motoi Kuwahara^2^, Miyuki Morikawa-Urase^2^,

Susumu Kusunoki^2^, and Ikuo Tsunoda^1*^

Departments of ^1^Microbiology and ^2^Neurology

Kindai University Faculty of Medicine

377-2 Ohnohigashi, Osakasayama, Osaka 589-8511 Japan

^3^Department of Psychology, Faculty of Sociology, Kansai University

3-3-35 Yamate-cho, Suita, Osaka 564-8680 Japan

**Table of contents**

**Figure S1**. Carbohydrate structures of 10 glycolipids and *k*-means clustering of glycoarray data ……………………………………………………………………... 3

**Figure S2**. Exploratory factor analysis (EFA) of IgG glycoarray data from Guillain-Barré syndrome (GBS) patients, excluding the patients with “Other GBS” …..... 4

**Tables S1-S12** ……………………………..................…..... Supplemental Tables.xlsx

**Table S1**. Anti-glycolipid IgG titers in sera from GBS patients

**Table S2**. Anti-glycolipid IgM titers in sera from GBS patients

**Table S3**. Factor scores of IgG glycoarray data clustered by *k*-means clustering and clinical information of 100 GBS patients

**Table S4**. Factor loadings of four factors in EFA of IgG glycoarray data

**Table S5**. Factor loadings of five factors in EFA of IgG glycoarray data

**Table S6**. Factor loadings of six factors in EFA of IgG glycoarray data

**Table S7**. Receiver Operating Characteristic (ROC) analysis between IgG factor scores and clinical data

**Table S8**. Factor loadings of three factors in EFA of IgM glycoarray data

**Table S9**. Factor loadings of four factors in EFA of IgM glycoarray data

**Table S10**. Factor scores of IgM glycoarray data clustered by *k*-means clustering and clinical information of 100 GBS patients

**Table S11**. ROC analysis between IgM factor scores and clinical data

**Table S12**. Factor loadings of five factors in EFA of IgG glycoarray data, excluding “Other GBS”

**
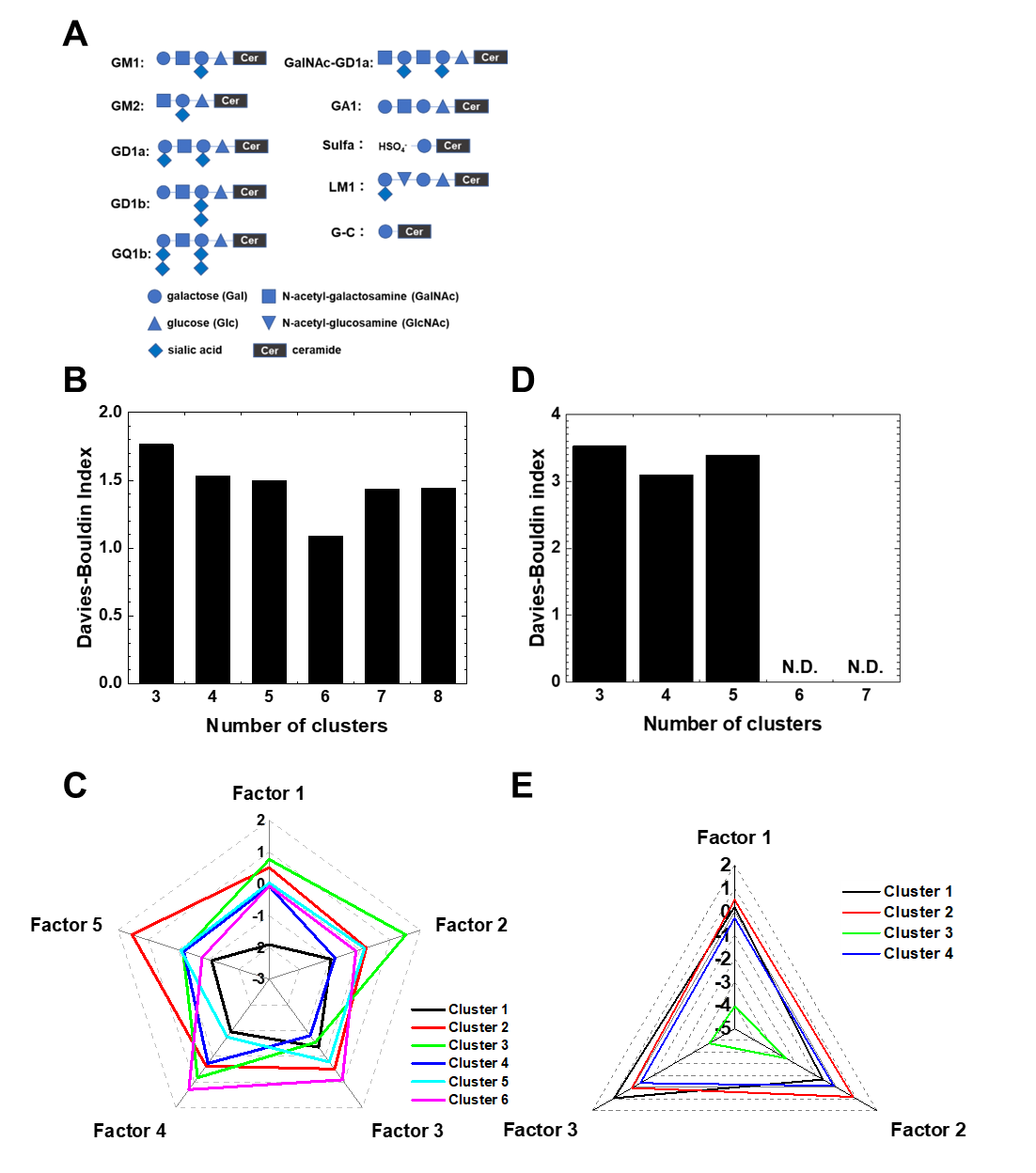
**

**d**

**b**

**a**

**e**

**c**

**Figure S1.** a) Carbohydrate structures of 10 glycolipids used in combinatorial glycoarray. Carbohydrate chains of glycolipids composed of galactose (Gal, ●), N-acetyl-galactosamine (GalNAc, ■), glucose (▲), N-acetyl-glucosamine (▼), and/or sialic acid (♦), are attached to ceramide (Cer). In glycoarray, we titrated antibodies against 10 glycolipids and 45 glycolipid complexes made of a combination of the two different glycolipids. Abbreviations: GM1, monosialo-tetrahexosyl-ganglioside; GM2, monosialo-trihexosyl-ganglioside; GD1, disialo-tetrahexosyl-ganglioside; GQ1b, tetrasialo-tetrahexosyl-ganglioside; GalNAc-GD1a, N-acetyl-galactosamine-GD1a; GA1, asialo-GM1; Sulfa, sulfatide; LM1, sialosyl-neolacto-tetraosyl-ceramide; and G-C, galactocerebroside. b, d) Davies-Bouldin (DB) index to determine the cluster number in *k*-means clustering of factor scores calculated from IgG (b) and IgM (d) glycoarray data of Guillain-Barré syndrome (GBS) patients. DB index indicated that the appropriate numbers of clusters are six in IgG data and four in IgM data. N.D.: we could not determine the index values because of convergence error. c, e) The score pattern of the centroid in each cluster was shown in a radar chart (c, IgG; e, IgM).


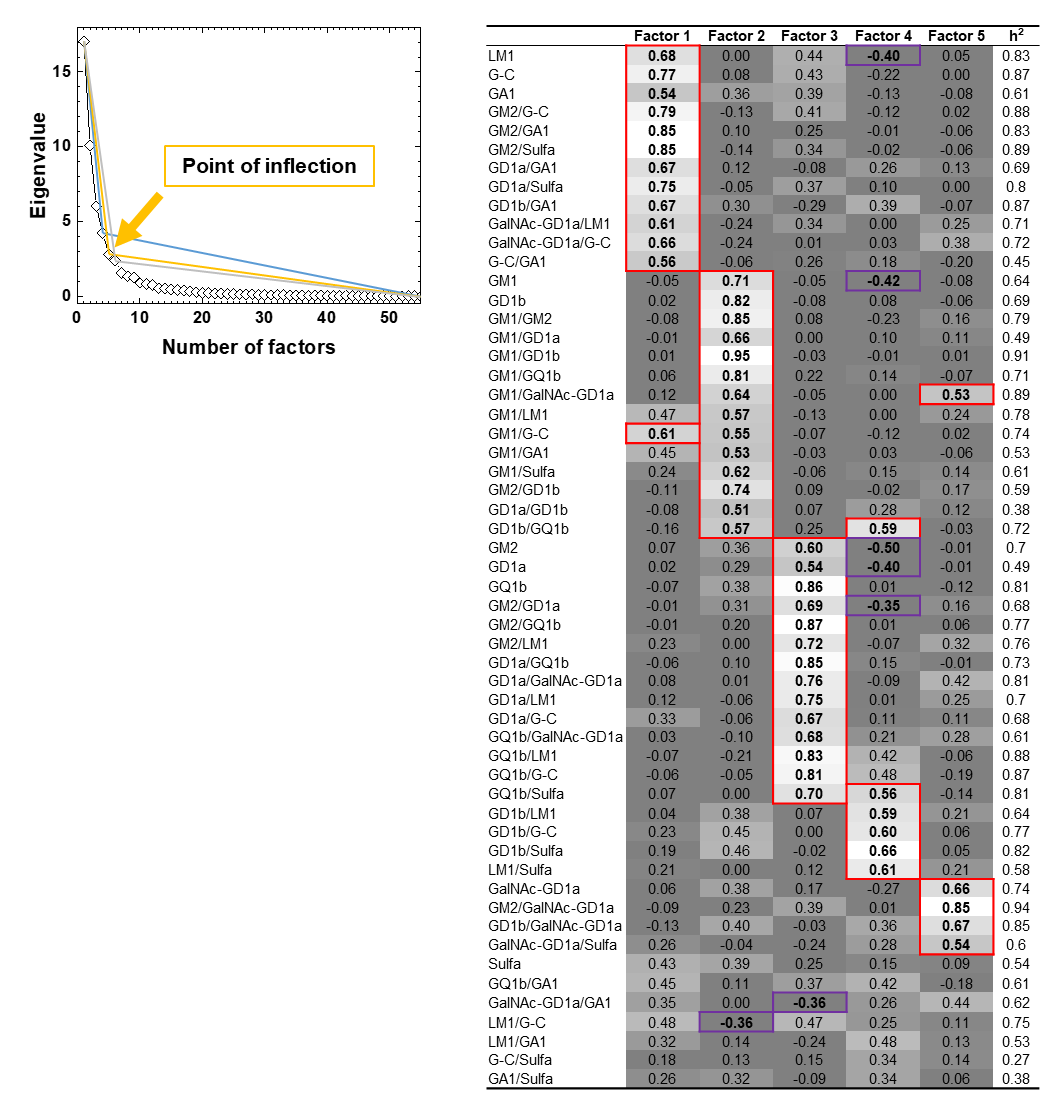


**a**

**b**

**Figure S2.** Exploratory factor analysis (EFA) of IgG glycoarray data from GBS patients, excluding the patients with “Other GBS.” a) The point of inflection of the scree plot with the eigenvalues and number of factors indicated that four, five, or six factors were the adequate number of common factors. b) Factor loadings higher than 0.5 (shown in red boxes) were selected as related variables forming the particular factors. Composition of each factor was similar to the case of the patients including “Other GBS.” A parameter, h^2^, indicates communality that is the ratio (0 to 1) of variance explained by the five Factors.
